# *Staphylococcus pseudintermedius* and *Staphylococcus schleiferi* Subspecies *coagulans* from Canine Pyoderma Cases in Grenada, West Indies, and Their Susceptibility to Beta-Lactam Drugs

**DOI:** 10.1155/2014/850126

**Published:** 2014-01-23

**Authors:** Harry Hariharan, Kathryn Gibson, Ross Peterson, Matthew Frankie, Vanessa Matthew, Joshua Daniels, Nancy A. Martin, Linton Andrews, Tara Paterson, Ravindra N. Sharma

**Affiliations:** ^1^Pathobiology Academic Program, St. George's University School of Veterinary Medicine, University Centre, Grenada; ^2^Department of Veterinary Clinical Sciences, Ohio State University School of Veterinary Medicine, Columbus, OH 43210, USA; ^3^Private Veterinary Practice, St. George, Grenada; ^4^Small Animal Medicine and Surgery Academic Program, St. George's University School of Veterinary Medicine, University Centre, Grenada

## Abstract

Over a 2-year period 66 cases of canine pyoderma in Grenada, West Indies, were examined by aerobic culture in order to ascertain the bacteria involved and their antimicrobial resistance patterns. Of the 116 total bacterial isolates obtained, the majority belonged to Gram-positive species, and the most common organism identified through biochemical and molecular methods was *Staphylococcus pseudintermedius*. Additionally, identification of a *Staphylococcus schleiferi* subspecies *coagulans* isolate was confirmed by molecular methods. All isolates of staphylococci were susceptible to beta-lactam drugs: amoxicillin-clavulanic acid, cefovecin, cefoxitin, cefpodoxime, and cephalothin. They were also susceptible to chloramphenicol and enrofloxacin. Resistance was highest to tetracycline. Methicillin resistance was not detected in any isolate of *S. pseudintermedius* or in *S. schleiferi*. Among the Gram-negative bacteria, the most common species was *Klebsiella pneumoniae*, followed by *Acinetobacter baumannii/calcoaceticus*. The only drug to which all Gram-negative isolates were susceptible was enrofloxacin. This report is the first to confirm the presence of *S. pseudintermedius* and *S. schleiferi* subspecies *coagulans*, in dogs with pyoderma in Grenada, and the susceptibility of staphylococcal isolates to the majority of beta-lactam drugs used in veterinary practice.

## 1. Introduction

Pyoderma, or bacterial infection of the skin, is the most common dermatologic problem encountered in dogs [1]. Varieties include superficial pyoderma, characterized by pustules, papules, erythema, focal crusting, and pruritus and deep pyoderma with furuncles and draining tracts. Although Gram-positive bacteria, such as staphylococci, are commonly involved in superficial pyoderma, Gram-negative bacteria can cause secondary infection, particularly in cases of deep pyoderma [2]. Empirical diagnosis of pyoderma based on history and physical examination is followed by complimentary tests, such as Gram staining and culture, with the most reliable results obtained from analysis of contents from an intact pustule [3]. Treatment usually involves antimicrobial drug therapy. Culture and antibiotic susceptibility testing are indicated in cases which do not respond to preliminary treatment and mandatory when treating deep pyoderma [4]. In addition, information on the principal organisms associated with pyoderma and their susceptibility patterns against commonly used antimicrobial drugs is highly useful in empirical treatment. The most commonly used drugs for empirical systemic treatment of canine pyoderma are amoxicillin-clavulanic acid and cephalosporins [5].

Until recently, *Staphylococcus intermedius* was considered the most common organism associated with canine pyoderma [3]. In actuality, isolates originally phenotypically identified as *S. intermedius* could be from three different species: *S. intermedius*, *Staphylococcus pseudintermedius*, and *Staphylococcus delphini *[6]. For definitive identification of these species, molecular diagnostic methods, such as polymerase chain reaction (PCR) techniques, are required [7]. Given the variety of sources of dogs, ranging from local Pompeks and Pothounds originating on the island to pets brought to the island from multiple locations in the USA, it was vital to study the bacteria from cases of canine pyoderma and their drug resistance patterns in Grenada. The resulting data on pathogens and drug resistance will form a background for establishment of recommendations for prudent use of antimicrobials for companion animals [8, 9].

The objectives of this study were to identify the bacteria associated with pyoderma in domestic dogs in Grenada, particularly with regard to the occurrence of *S. pseudintermedius* and the emerging pathogen *Staphylococcus schleiferi*, and to determine the extent of antimicrobial drug resistance. Although it was hypothesized that the majority of pyoderma cases would be due to staphylococci, occurrence of other Gram-positive and Gram-negative species and their antimicrobial resistance patterns were also studied. Results from these data are likely to assist towards selection of appropriate antimicrobial treatment plans.

## 2. Materials and Methods

### 2.1. Sample Collection

Samples were obtained from 66 domestic dogs diagnosed with pyoderma between March, 2010, and February, 2012. All cases presented during the 2-year period were included in the study. The dogs were diagnosed at St. George's University Small Animal Hospital, the Grenada Society for the Prevention of Cruelty to Animals (GSPCA), and one other veterinary practice in the St. George's parish on Grenada, West Indies. All dogs were client owned, and their ages varied from 6 weeks to 14 years, with the average being 2.6 years of age. The majority of the dogs were of mixed breed (48/66). Others included Pompek (6), Pothound (3), Pit bull (2), and one each of 7 breeds/types. Pustule contents or swabs applied to ulcerated lesions were obtained aseptically, using Carry Blair Transport Swabs (Becton, Dickinson and Company, Sparks, MD, USA). Transport swabs were stored at 4°C immediately after collection and were cultured within 24 h.

### 2.2. Bacterial Culture

All samples were plated on blood and MacConkey agar and incubated aerobically at 37°C for up to 72 h. Culture plates were examined daily for the number and types of colonies. Samples with 2 colonies or fewer after 72 h were considered negative. Bacterial growth was classified as pure or mixed. Bacteria were initially identified based on colony morphology; Gram stain; and other preliminary testing methods, including catalase, coagulase, and oxidase tests [10]. Further classification of bacteria involved the use of API bacterial identification strips (bioMérieux Inc., Durham, NC, USA), multiplex PCR, and sequencing. API strips were not able to distinguish among *S. intermedius*, *S. pseudintermedius*, and *S. delphini*.

### 2.3. DNA Isolation

Bacterial DNA from all isolates phenotypically identified as *S. intermedius* was extracted using the DNeasy Blood & Tissue kit (Qiagen, Valencia, CA, USA) and the manufacturer's Gram-positive protocol. As a modification, preliminary incubation was performed in enzymatic lysis buffer, consisting of 20 mg/mL lysozyme (Sigma-Aldrich, St. Louis, MO, USA) and 1.2% Triton X-100 (Sigma) in phosphate-buffered saline, pH 7.4. DNA content was checked by a NanoDrop2000C (Thermo Scientific, Wilmington, DE, USA). DNA was forwarded to the Veterinary Teaching Hospital Microbiology Laboratory at The Ohio State University College of Veterinary Medicine, Columbus, OH, USA.

### 2.4. Multiplex PCR

Multiplex PCR was performed as previously described [11]. Multiplex PCR utilized Invitrogen Platinum Mastermix (Life Technologies, Grand Island, NY, USA) in 25 µL total volume reactions. Controls for multiplex PCR included *Staphylococcus aureus* (ATCC 29213) (American Type Culture Collection, Manassas, VA, USA), *S. pseudintermedius* (LMG 22221) (LMG Bacteria Collection, Laboratory of Microbiology, Gent, Belgium), and *S. intermedius* (ATCC 29663). The result for *S. schleiferi* subspecies *coagulans* was based on comparison of the band size relative to the DNA ladder and other control staphylococci.

### 2.5. Sequencing of the *rpoB* Gene Fragment

For confirmation of the identification of *S. schleiferi* subsp. *coagulans, *the single isolate was tested by sequencing a fragment of the *rpoB *gene as previously described [12]. This method was additionally applied to just one isolate originally identified as *S. intermedius* by API strip, but identified as *S. aureus *by multiplex PCR. Sequencing results were identified using the National Center for Biotechnology Information Basic Local Alignment Search Tool (BLAST) against highly similar sequences (megablast) and protein-protein BLAST (blastp) using the corresponding databases.

### 2.6. Antimicrobial Susceptibility Testing

Antimicrobial susceptibility tests were performed on isolates using the disk diffusion method on Mueller-Hinton agar, as recommended by the Clinical and Laboratory Standards Institute (CLSI), and the zone sizes were interpreted per CLSI guidelines [13]. Antimicrobial drugs used against Gram-positive isolates, and the disk potencies were as follows: amoxicillin-clavulanic acid (30 µg), ampicillin (10 µg), cefovecin (30 µg), cefpodoxime (10 µg), cefoxitin (30 µg), cephalothin (30 µg), chloramphenicol (30 µg), clindamycin (2 µg), enrofloxacin (5 µg), erythromycin (30 µg), gentamicin (10 µg), neomycin (30 µg), penicillin (10 units), sulfamethoxazole-trimethoprim (25 µg), and tetracycline (30 µg). For enrofloxacin, zone sizes were interpreted per CLSI guidelines for bacteria from animals [14]. All antimicrobial disks, except cefovecin were obtained from Becton, Dickinson, and Company. Cefovecin disks were obtained from Pfizer Animal Health (New York City, NY, USA). As per the manufacturer's instructions, isolates giving growth inhibition zone diameters less than or equal to 19 mm with cefovecin were considered resistant. Gram-negative isolates were tested only against amoxicillin-clavulanic acid, ampicillin, cephalothin, enrofloxacin, gentamicin, neomycin, sulfamethoxazole-trimethoprim, and tetracycline, and the disk potencies were the same as indicated above.

All *S. pseudintermedius* and the single *S. schleiferi* subsp. *coagulans* isolates were tested for methicillin resistance using 2 methods. A zone of growth inhibition of ≤17 mm against an oxacillin 1 µg disk was considered indicative of resistance as recommended by Bemis et al. [15] and Gold et al. [16]. Growth on MRSA ID chromagar (bioMérieux) was the second test used as per the recommended by Horstmann et al. [17]. The test was conducted as per the manufacturer's directions and as outlined by Diederen et al. [18].

### 2.7. History of Antimicrobial Use

Histories of previous antimicrobial use for pyoderma cases were collected when these were available.

## 3. Results

### 3.1. Bacterial Identification

A total of 116 bacterial isolates were obtained from the 66 canine pyoderma cases. The number of isolates per case ranged from 1 colony type (pure culture) to 4 colony types (mixed infection). Twenty-one of the 66 cases (31.8%) yielded pure culture. Of the total 116 isolates, 62 (53.5%) were Gram-positive, and 54 (46.5%) were Gram-negative. Distribution of bacterial isolates is provided in Table 1.

Staphylococci were the most commonly isolated Gram-positive bacteria. Of the 43 isolates of staphylococci, 18 (41.9%) were from cases yielding a pure culture and 25 (58.1%) were from mixed infections. Using multiplex PCR, 27 of the 29 isolates identified by API strips as *S. pseudintermedius* were identified as *S. pseudintermedius*, 1 as *S. aureus*, and 1 as *S. schleiferi* subsp. *coagulans*. The isolate identified as *S. aureus*, which had phenotypic properties of *S. intermedius,* was subjected to *rpoB* gene sequencing, and it was identified as *S. pseudintermedius*. The identity of the *S. schleiferi* subsp. *coagulans* isolate was also confirmed by sequencing. Of the 28 *S. pseudintermedius* isolates, 11 (39.3%) were obtained in pure culture, and 17 (60.7%) were from mixed infections.

Regarding the remaining 34 Gram-positive isolates, there were 6 *Staphylococcus hominis*, 2 *Staphylococcus lugdunensis*, 1 *Staphylococcus capitis*, 1 *Staphylococcus epidermidis*, 1 *Staphylococcus xylosus*, and 3 unspeciated *Staphylococcus*. Other Gram-positive isolates included 6 speciated and 5 unspeciated streptococci, 3 *Corynebacterium* spp., 3 *Micrococcus* spp., and 2 *Bacillus* spp. Seven (20.6%) of these 34 Gram-positive isolates were recovered in pure culture, including 3 *S. hominis* and 2 *S. lugdunensis*. All streptococci, *Corynebacterium* spp., *Micrococcus* spp., and *Bacillus* isolates were from mixed cultures.

Of the 54 Gram-negative isolates, the most frequent was *Klebsiella pneumoniae *(9 isolates), followed by 8 *A. baumannii*/*calcoaceticus*, 7 *Escherichia coli*, 6 *Pseudomonas aeruginosa*, 5 *Enterobacter cloacae*, and 5 *Proteus mirabilis*. There were 2 or fewer isolates of the remaining Gram-negative species, including 2 *Pseudomonas oryzihabitans*, and 2 *Pantoea* spp. All Gram-negative isolates were recovered from cases with mixed infection, except for 1 *A. baumannii/calcoaceticus* and 2 *Proteus mirabilis*.

### 3.2. Antimicrobial Drug Susceptibility

All isolates of staphylococci, streptococci, and the 6 major Gram-negative species (consisting of *K. pneumoniae*, *A. baumannii*/*calcoaceticus*, *E. coli*, *P. aeruginosa*, *P. mirabilis*, and *E. cloacae*) were tested for antimicrobial susceptibility. All tested bacteria were susceptible to enrofloxacin, and all 43 isolates of staphylococci were susceptible to beta-lactam drugs: amoxicillin-clavulanic acid, cefovecin, cefoxitin, cefpodoxime, and cephalothin (Table 2). In addition, all isolates of staphylococci were susceptible to chloramphenicol. *Staphylococcus lugdunensis*, *S. capitis*, *S. epidermidis*, *S. schleiferi* subsp. *coagulans*, and *S*. *xylosus* were susceptible to all 15 drugs. Staphylococci were most frequently resistant to tetracycline (30.2%), followed by sulfamethoxazole-trimethoprim (14%); penicillin (11.6%); clindamycin, erythromycin, gentamicin, and neomycin (7% each); and ampicillin (2.3%). Multidrug resistance was evident only among *S. pseudintermedius *isolates (6/28) and *S. hominis* isolates (3/6).

Methicillin resistance was not detected in any of the 29 isolates of staphylococci tested (28 *S. pseudintermedius* and one *S*. *schleiferi*). Among the 11 isolates of streptococci, resistance was seen most frequently against neomycin (100%), followed by tetracycline (54.5%), sulfamethoxazole-trimethoprim (27.3%), and gentamicin (18.2%).

Of the 40 Gram-negative isolates tested against 8 drugs, resistance was most frequent to ampicillin (75%), followed by cephalothin (57.5%), tetracycline and amoxicillin-clavulanic acid (35% each), gentamicin and neomycin (12.5% each), and sulfamethaxole-trimethoprim (5%). Multidrug resistance was seen most commonly in *P. aeruginosa* (100%), with characteristic resistance to ampicillin, amoxicillin-clavulanic acid, and trimethoprim-sulfa, followed by *A. baumannii*/*calcoaceticus* (62.5%). Multidrug resistance was not seen among the *E. coli* isolates.

Only 9 dogs with pyoderma (13.6%) had histories of previous antimicrobial use. Four of five dogs with histories of previous treatment with cephalexin yielded *S. pseudintermedius*, and the fifth had *Streptococcus dysgalactiae*, and all were susceptible to cephalothin and other cephalosporins. Two dogs had prior treatment with lincomycin, and the staphylococcal isolates from these showed no resistance to cephalosporins, but one isolate was resistant to clindamycin. Of the remaining 2 dogs, one was treated earlier with tetracycline, and it was positive for tetracycline-resistant *S. pseudintermedius*, and the other treated with amoxicillin-clavulanic acid yielded only *S. pseudintermedius* susceptible to all drugs.

## 4. Discussion

It has been demonstrated that phenotypically identified *S. intermedius* strains could include not only true *S. intermedius* strains, but also 2 other species in this group, namely, *S. pseudintermedius* and *S. delphini*. In fact, most of the canine strains of this group have been identified as *S. pseudintermedius*, not *S. intermedius *[6, 7]. *S. pseudintermedius* was the predominant isolate from 66 cases of canine pyoderma in the present study. This is in accordance with the recent findings from a study in Japan [19], in which none of the 190 isolates from canine pyoderma cases belonged to *S. intermedius *or *S. aureus* species.

In the present study, a single isolate phenotypically identified by API strip as *S. intermedius* was identified by both multiplex PCR and DNA sequencing as *S. schleiferi* subsp. *coagulans*. Historically, the early isolations of *S. schleiferi* were from a canine otitis case in Japan in 1990 [20] and 2 canine pyoderma cases in Europe in 2002 [21]. Since then, isolation of this *staphylococcus* from dogs has been reported in other countries including USA, and *S. schleiferi *has been noted to occur in recurring pyoderma cases, dogs with both otitis and pyoderma, and apparently healthy dogs [22]. Recent findings suggest that the 2 subspecies: *S. schleiferi *subsp. *coagulans* (coagulase positive) and subsp. *schleiferi* (coagulase negative) are not genetically distinct and are likely variations of coagulase-producing strains within one species [23].

In this study, 11.6% of staphylococci showed resistance to penicillin, whereas resistance to ampicillin was only 2.3%. In a recent study [24], of 67 *S*. *pseudintermedius* isolates form dogs, penicillin resistance was 61%, whereas ampicillin resistance was 40%. Methicillin resistance was not tested in their study, but all clinical isolates were included. It is possible that resistance mechanisms other than beta-lactamase production may play a role. Modification of penicillin-binding proteins is increasingly important as another mechanism of resistance to penicillins [25]. Further studies are needed to elucidate the mechanisms involved. The antibiotics of choice for extended therapy in canine pyoderma are cephalexin, amoxicillin-clavulanic acid, and enrofloxacin [1]. All 28 *S. pseudintermedius* isolates in the present study were susceptible to amoxicillin-clavulanic acid, cephalosporins, including cephalothin (which indicates susceptibility to cephalexin), and enrofloxacin. Cephalexin is bactericidal, has a low potential for development of resistance, and has minimal side effects [2]. Cephalexin and cefpodoxime are the most commonly prescribed drugs for *S. pseudintermedius* pyoderma in dogs [26]. The usage of cephalexin is justifiable as long as pyoderma is not complicated by Gram-negative bacteria. Moreover, the limited data in the present study on the history of antimicrobial use also showed susceptibility of all isolates from the treated animals to cephalosporins. It is known that canine pyoderma may be complicated by Gram-negative organisms such as *E. coli, Proteus* spp., and *Pseudomonas* spp. [3]. The role breeds of dogs and high humidity and heat on the role of Gram-negative bacteria in canine pyoderma may be worth investigating. Breed may be relevant in the pathogenesis of canine pyoderma [27]. Nearly 58% of the 6 most commonly-occurring Gram-negative species in the present study were resistant to cephalexin. Whether or not cephalothin-resistant Gram-negative bacteria are susceptible to other cephalosporins, such as cefovecin is worth investigating in future studies. In a recent study [28], cefovecin was found to have excellent *in-vitro* activity against a variety of both Gram-positive and Gram-negative pathogens from dogs.

Enrofloxacin was effective against all tested bacterial isolates in the present study. Both enrofloxacin and orbifloxacin, 2 fluoroquinolone drugs with similar antimicrobial activity, are safe and effective in treating superficial and deep pyoderma in dogs [29]. Fluoroquinolones have modest immunomodulating properties as well [30]. In a study published in 2006 from Canada, only 1% of 651 *S. intermedius* isolates from canine otitis were resistant to enrofloxacin [31].

Isolates resistant to clindamycin, another drug used for treatment of staphylococcal infections in dogs [32], were found in the present study. Same was the case with erythromycin. The macrolide antibiotics erythromycin and tylosin have been used to treat pyoderma cases [33].

## 5. Conclusions

This report is the first to confirm the presence of *S. pseudintermedius* and *S. schleiferi *subsp. *coagulans *in dogs with pyoderma in Grenada, and the susceptibility of staphylococcal isolates to the majority of beta-lactam drugs used in veterinary practice. Nearly a quarter of the *S. pseudintermedius* isolates in the present study showed resistance to more than one drug. Monitoring drug resistance trends is essential as new drug-resistant strains from other parts of the world could be introduced in dogs located in the island nation of Grenada. It is also important to monitor emergence of *S. schleiferi *and other new species, as well as beta-lactam resistance in canine strains of staphylococci.

## Figures and Tables

**Table 1:** Types and number of bacterial isolates from canine pyoderma cases in Grenada, West Indies.

Isolate identification	No. (%) of isolates (*n* = 116)	No. (%) of pure culture cases (*n* = 66)^a^
Gram-positive isolates	62 (53.5)	18 (27.3)
Staphylococci	43 (37.1)	18 (27.3)
* Staphylococcus pseudintermedius *	28 (24.1)	11 (16.7)
* Staphylococcus hominis *	6 (5.2)	3 (4.6)
* Staphylococcus lugdunensis *	2 (1.7)	2 (3.0)
* Staphylococcus schleiferi subspecies coagulans *	1 (0.9)	0 (0.0)
* Staphylococcus capitis *	1 (0.9)	0 (0.0)
* Staphylococcus epidermidis *	1 (0.9)	1 (1.5)
* Staphylococcus xylosus *	1 (0.9)	1 (1.5)
Unspeciated staphylococci	3 (2.6)	0 (0.0)
Streptococci	11(9.5)	0 (0.0)
* Streptococcus dysgalactiae *	4 (3.5)	0 (0.0)
* Streptococcus agalactiae *	1 (0.9)	0 (0.0)
* Streptococcus canis *	1 (0.9)	0 (0.0)
Unspeciated streptococci	5 (4.3)	0 (0.0)
* Corynebacterium* spp.	3 (2.6)	0 (0.0)
* Micrococcus* spp.	3 (2.6)	0 (0.0)
* Bacillus* spp.	2 (1.7)	0 (0.0)
Gram-negative isolates	54 (46.5)	3 (4.5)
* Klebsiella pneumoniae *	9 (7.8)	0 (0.0)
* Acinetobacter baumannii/calcoaceticus *	8 (6.9)	1 (1.5)
* Escherichia coli *	7 (6.0)	0 (0.0)
* Pseudomonas aeruginosa *	6 (5.2)	0 (0.0)
* Enterobacter cloacae *	5 (4.3)	0 (0.0)
* Proteus mirabilis *	5 (4.3)	2 (3.0)
Other species^b^	14 (12.1)	0 (0.0)

^a^indicates the number of canine pyoderma cases in which the given type of bacterial isolate was found in pure culture. *n*: total number of canine pyoderma cases.

^b^includes the following isolates: *Pseudomonas oryzihabitans* and *Pantoea* spp. (2 each) and *Acinetobacter* spp., *Chryseobacterium indologenes*, *Escherichia hermannii*, *Enterobacter sakazakii*, *Moraxella nonliquefaciens*, *Proteus vulgaris*, *Pseudomonas fluorescens*, *Pseudomonas* spp., *Raoultella terrigena*, and *Stenotrophomonas maltophilia*, (1 each).

**Table 2:** Antimicrobial resistance patterns of staphylococci from canine pyoderma cases in Grenada, West Indies.

Antimicrobial drug resistance	No. (%) of *S. pseudintermedius* (*n* = 28)	No. (%) of *S. hominis* (*n* = 6)	No. (%) of other staphylococci^a^ (*n* = 9)	No. (%) total (*n* = 43)
Beta-lactams^b^				
Amoxicillin-clavulanic acid	0 (0.0)	0 (0.0)	0 (0.0)	0 (0.0)
Cefovecin	0 (0.0)	0 (0.0)	0 (0.0)	0 (0.0)
Cefoxitin	0 (0.0)	0 (0.0)	0 (0.0)	0 (0.0)
Cefpodoxime	0 (0.0)	0 (0.0)	0 (0.0)	0 (0.0)
Cephalothin	0 (0.0)	0 (0.0)	0 (0.0)	0 (0.0)
Ampicillin	1 (3.6)	0 (0.0)	0 (0.0)	1 (2.3)
Penicillin	4 (14.3)	1 (14.3)	0 (0.0)	5 (11.6)
Other drugs				
Chloramphenicol	0 (0.0)	0 (0.0)	0 (0.0)	0 (0.0)
Enrofloxacin	0 (0.0)	0 (0.0)	0 (0.0)	0 (0.0)
Clindamycin	1 (3.6)	2 (33.3)	0 (0.0)	3 (7.0)
Erythromycin	1 (3.6)	2 (33.3)	0 (0.0)	3 (7.0)
Gentamicin	1 (3.6)	2 (33.3)	0 (0.0)	3 (7.0)
Neomycin	1 (3.6)	2 (33.3)	0 (0.0)	3 (7.0)
Sulfamethoxazole-trimethoprim	4 (14.3)	2 (28.6)	0 (0.0)	6 (14.0)
Tetracycline	9 (32.1)	4 (57.1)	0 (0.0)	13 (30.2)

^a^includes the following isolates: *S. lugdunensis *(2); *S. capitis*, *S. epidermidis*, *S. schleiferi* subsp. *coagulans*, and *S. xylosus* (1 each); and unspeciated staphylococci (3).

^b^Methicillin resistance (tested with oxacillin disk and a chromogenic medium) was not detected in any of the *S. pseudintermedius *or *S. schleiferi* isolates.
